# How important is randomisation in a stepped wedge trial?

**DOI:** 10.1186/s13063-015-0872-1

**Published:** 2015-08-16

**Authors:** James R Hargreaves, Audrey Prost, Katherine L. Fielding, Andrew J. Copas

**Affiliations:** Department of Social and Environmental Health Research, London School of Hygiene and Tropical Medicine, 15-17 Tavistock Place, London, WC1H 9SH UK; Institute for Global Health, University College London, Gower St, London, WC1E 6BT UK; Department of Infectious Disease Epidemiology, London School of Hygiene and Tropical Medicine, Keppel St, London, WC1E 7HT UK; Medical Research Council Clinical Trials Unit at University College, 125 Kingsway, London, WC2B 6NH UK

## Abstract

In cluster randomised trials, randomisation increases internal study validity. If enough clusters are randomised, an unadjusted analysis should be unbiased. If a smaller number of clusters are included, stratified or matched randomisation can increase comparability between trial arms. In addition, an adjusted analysis may be required; nevertheless, randomisation removes the possibility for systematically biased allocation and increases transparency. In stepped wedge trials, clusters are randomised to receive an intervention at different start times (‘steps’), and all clusters eventually receive it. In a recent study protocol for a ‘modified stepped wedge trial’, the investigators considered randomisation of the clusters (hospital wards), but decided against it for ethical and logistical reasons, and under the assumption that it would not add much to the rigour of the evaluation. We show that the benefits of randomisation for cluster randomised trials also apply to stepped wedge trials. The biggest additional issue for stepped wedge trials in relation to parallel cluster randomised trials is the need to control for secular trends in the outcome. Analysis of stepped wedge trials can in theory be based on ‘horizontal’ or ‘vertical’ comparisons. Horizontal comparisons are based on measurements taken before and after the intervention is introduced in each cluster, and are unbiased if there are no secular trends. Vertical comparisons are based on outcome measurements from clusters that have switched to the intervention condition and those from clusters that have yet to switch, and are unbiased under randomisation since at any time point, which clusters are in intervention and control conditions will have been determined at random. Secular outcome trends are a possibility in many settings. Many stepped wedge trials are analysed with a mixed model, including a random effect for cluster and fixed effects for time period to account for secular trends, thereby combining both vertical and horizontal comparisons of intervention and control clusters. The importance of randomisation in a stepped wedge trial is that the effects of time can be estimated from the data, and bias from secular trends that would otherwise arise can be controlled for, provided the trends are correctly specified in the model.

## Background

In stepped wedge trials, clusters are randomised to receive an intervention at different start times (‘steps’), and all clusters eventually receive it. The popularity of the design is increasing and debate about their use, design and analysis is ongoing [1–3]. This issue of *Trials* features a protocol for a study described as a ‘modified stepped wedge’ [4]. DiDiodato *et al*. investigate whether an antimicrobial stewardship intervention involving prospective chart audit and feedback could reduce the length of stay in hospital for patients admitted with community-acquired pneumonia [4]. They plan to introduce the intervention in four hospital wards sequentially, but without randomising the order of introduction. Importantly, the authors state that they considered randomisation but decided against it for ethical and logistical reasons, and under the assumption that it would not add much to the rigour of the evaluation. In this commentary we examine the value of randomisation in stepped wedge trials, provide a brief critical appraisal of DiDiodato *et al*.’s argument concerning the (lack of) value of randomisation in their own study, and close with a summary of arguments in favour of randomisation in stepped wedge trials.

## Main Text

### Why is randomisation useful in stepped wedge trials?

Randomisation brings several important benefits to cluster randomised controlled trials. Most importantly, randomisation increases the internal validity of the study. Analyses of cluster randomised controlled trials generally compare outcomes between intervention and control groups after some post-randomisation follow-up period. If enough clusters are randomised, an unadjusted comparison should be unbiased. In some cases, especially in cluster randomised controlled trials with relatively few clusters, adjustment for potentially imbalanced covariates is included in the primary analysis [5]. Cluster randomised controlled trials can also make use of stratified or restricted randomisation to create more comparable trial arms. Finally, randomisation can help to increase the transparency and perceived fairness of allocation. All of these features are also valuable in stepped wedge trials.

The biggest additional issue for stepped wedge trials in relation to parallel cluster randomised trials is the need to control for secular trends in the outcome of interest. Analysis of stepped wedge trials can in theory be based on ‘horizontal’ or ‘vertical’ comparisons or, as is most common in practice, a mixture of the two. In their simplest form horizontal comparisons are based on measurements taken before and after the intervention is introduced in each cluster. By contrast, vertical comparisons are based on outcome measurements from clusters that have switched to the intervention condition and those from clusters that have yet to switch, within each of several time intervals which may correspond to the steps of the design.

Horizontal, or before-after, comparisons are unbiased if there are no secular trends. However, secular outcome trends are a possibility in many settings. A key importance of randomisation is to allow the estimation of an unbiased intervention effect even when there are secular trends. Within a stepped wedge trial, vertical comparisons are unbiased under randomisation since at any time point, which clusters are in the intervention and control conditions will have been determined at random. Within a stepped wedge trial randomisation also allows secular trends to be estimated from the data. If, conversely, clusters are allocated to groups to receive the intervention at certain times in a systematic way, such as clusters likely to have poor outcomes receive intervention first, then secular trends cannot generally be distinguished from the effects of this allocation mechanism.

In practice, most stepped wedge trials are analysed using a mixed approach that uses all the data in a single analysis stage, combining both vertical and horizontal comparisons of intervention and control clusters [6]. For example a mixed model may be fitted, including a random effect for each cluster and fixed effects for time period to account for secular trends. The importance of randomisation is that these effects of time can be estimated from the data, and hence bias from secular trends that would otherwise arise can be controlled for, at least provided the trends are correctly specified in the model. We have argued in favour of careful modelling of time trends, and cross-checking results from mixed approaches with estimates from vertical approaches that do not require the form of time trends to be specified [6, 7].

### What would DiDiodato *et al*. have gained from randomisation?

The study by DiDiodato *et al*. involves introducing an antimicrobial stewardship intervention in a non-random sequential manner to four hospital wards (clusters), to see whether it will reduce pneumonia patients’ length of stay in hospital. The main argument that the authors provide for the lack of benefit from randomisation of wards is that patients are allocated to these in a quasi-random fashion, based on bed availability. Furthermore, the hospitalists that manage patients work across all four wards. Though not stated explicitly, the authors’ argument suggests that wards are thought to be of little relevance to outcomes in the trial, and the sample size calculation ignores this level of clustering. If this were the case, rather than thinking of the study as including a cluster-level allocation mechanism, the study could rather be conceptualised as incorporating an individually quasi-randomised allocation, with an allocation ratio that varies over time from favouring control to later favouring intervention. If there are no ‘ward effects’ then the non-random ordering of the time at which the intervention is introduced would not present a risk of bias. However, the authors go on to propose an analysis that does incorporate fixed effects of wards, and of time from the start of the study, so it seems that both ward effects and secular trends are considered possible.

The authors obviously have excellent knowledge of the trial setting, but it seems plausible that the systematic allocation order of wards (by expected number of pneumonia patients) may prevent the unbiased estimation of any secular trends affecting all wards in the proposed analysis, and consequently the intervention effect may also be biased. This bias might have been avoided by randomising the wards, although a further issue in this trial is that the number of wards is very small. In the current design, bias in the intervention effect may still arise from secular trends, even if it is the case that the individual patient allocation to wards by hospitalists is indeed conducted in a fashion that mimics randomisation. In relation to this last point, however, we are additionally concerned that if the intervention is indeed successful in reducing length of hospital stay in the first cluster allocated, this has the potential to influence the subsequent allocation of patients to wards, since it is bed availability that is cited as the main quasi-random factor determining this allocation.

In our view, the study by DiDiodato *et al*. is not usefully classified as a modified stepped wedge trial, but rather as a form of before and after cross-over study conducted in four hospital wards. As such, its analysis raises the validity question presented by all before and after studies: could there be secular trends due to things influencing outcomes over the study period of 24 months other than the intervention? This seems plausible, casting doubts on the robustness of this design. In presenting their findings the authors will need to make a strong case against the potential presence of such effects.

## Conclusion

In stepped wedge trials, randomisation allows an unbiased analysis, subject to taking due care in appropriately specifying time-trends in the analysis. The DiDiodato *et al*. study protocol described in this issue of *Trials* is perhaps best conceptualised as a multi-ward, before-after study rather than a modified stepped wedge trial. More generally, the comparison between a non-randomised stepped wedge trial and a randomised stepped wedge trial is in essence the same as between any non-randomised allocation design and a cluster randomised controlled trial: the non-randomised design may give an unbiased answer, but it is difficult to know this. We therefore argue for randomisation in stepped wedge trials whenever it is logistically feasible.

## References

[1] Mdege ND, Man MS, Taylor Nee Brown CA, Torgerson DJ (2011). Systematic review of stepped wedge cluster randomized trials shows that design is particularly used to evaluate interventions during routine implementation. J Clin Epidemiol.

[2] Hemming K, Haines TP, Chilton PJ, Girling LJ, Lilford RJ (2015). The stepped wedge cluster randomised trial: rationale, design, analysis, and reporting. BMJ.

[3] Hargreaves J, Copas AJ, Beard E, Osrin D, Lewis JL, Davey C, et al. Five questions to consider before conducting a stepped wedge trial. Trials. 2015 [Under review]. 10.1186/s13063-015-0841-8 PMC4538743 26279013

[4] DiDiodato G, McArthur L, Beyene L, Smieja M, Thabane L. Evaluating the effectiveness of an antimicrobial stewardship program on reducing the length of stay of immune-competent adult patients admitted to a hospital ward with a diagnosis of community-acquired pneumonia: study protocol for a pragmatic clinical trial. Trials. 2015 [Under review]. 10.1186/s13063-015-0871-2 PMC4535257 26272324

[5] Hayes RJ, Moulton LH (2009). Cluster randomised trials.

[6] Davey C, Hargreaves J, Thompson J, Copas A, Beard E, Lewis JJ, Fielding K. Analysis and reporting of stepped wedge randomised-controlled trials: synthesis and critical appraisal of published studies, 2010–2014. Trials. 2015 [Under review]. 10.1186/s13063-015-0838-3 PMC4538923 26278667

[7] Copas A, Lewis JJ, Thompson JA, Davey C, Fielding K, Baio G, et al. Designing a stepped wedge trial: three main designs, carry-over effects and randomisation approaches. Trials. 2015 [Under review]. 10.1186/s13063-015-0842-7 PMC4538756 26279154

